# BO-CLAHE enhancing neonatal chest X-ray image quality for improved lesion classification

**DOI:** 10.1038/s41598-025-88451-0

**Published:** 2025-02-10

**Authors:** Jiwon Han, Byungmin Choi, Jae Young Kim, Yeonjoon Lee

**Affiliations:** 1 Department of Applied Artificial Intelligence, Hanyang University, Seoul, 15588 Korea; 2 Department of Pediatrics, Ansan Hospital, College of Medicine, Korea University, Ansan, 15355 Republic of Korea; 3 Department of Convergence Medicine, College of Medicine, Korea University, Seoul, 02841 Republic of Korea; 4 Department of Computer Science and Engineering, Hanyang University, Seoul, 15588 Republic of Korea

**Keywords:** Preterm, High-risk neonates, Neonatal chest X-ray, CLAHE, Bayesian optimization, Paediatric research, Translational research

## Abstract

In the case of neonates, especially low birth weight preterm and high-risk infants, portable X-rays are frequently used. However, the image quality of portable X-rays is significantly lower compared to standard adult or pediatric X-rays, leading to considerable challenges in identifying abnormalities. Although attempts have been made to introduce deep learning to address these image quality issues, the poor quality of the images themselves hinders the training of deep learning models, further emphasizing the need for image enhancement. Additionally, since neonates have a high cell division rate and are highly sensitive to radiation, increasing radiation exposure to improve image quality is not a viable solution. Therefore, it is crucial to enhance image quality through preprocessing before training deep learning models. While various image enhancement methods have been proposed, Contrast Limited Adaptive Histogram Equalization (CLAHE) has been recognized as an effective technique for contrast-based image improvement. However, despite extensive research, the process of setting CLAHE’s hyperparameters still relies on a brute force, manual approach, making it inefficient. To address this issue, we propose a method called Bayesian Optimization CLAHE(BO-CLAHE), which leverages Bayesian optimization to automatically select the optimal hyperparameters for X-ray images used in diagnosing lung diseases in preterm and high-risk neonates. The images enhanced by BO-CLAHE demonstrated superior performance across several classification models, with particularly notable improvements in diagnosing Transient Tachypnea of the Newborn (TTN). This approach not only reduces radiation exposure but also contributes to the development of AI-based diagnostic tools, playing a crucial role in the early diagnosis and treatment of preterm and high-risk neonates.

## Introduction

Low birth weight preterm and high-risk neonates face significant challenges when it comes to lesion classification due to several factors. First, there is a lack of X-ray data for these populations compared to adults, which limits the development of effective diagnostic tools. Second, the few available images often suffer from poor quality, making it difficult to distinguish lesions. Additionally, interpreting low-quality images is challenging for even the most skilled experts, and any delay in diagnosing chest diseases can lead to complications. As noted by Subramani et al.^1^, medical images frequently exhibit issues such as low contrast and poor visibility, which significantly hinder accurate diagnosis. Their study highlights the importance of advanced preprocessing techniques to enhance diagnostic details in such challenging scenarios. For these reasons, advanced techniques like deep learning are needed to aid in more accurate and timely diagnosis.

However, applying deep learning to classify lesions in preterm and high-risk neonates also presents challenges. Posterior-anterior (PA) and anterior-posterior (AP) imaging techniques are often impractical for these patients, leading to widespread use of portable imaging devices^2^. While these devices are essential for quick diagnosis, they come with a significant trade-off: reduced image quality. Enhancing the image quality is necessary, but due to neonates’ sensitivity to radiation, increasing radiation exposure to improve image quality is not a viable option. Therefore, there is a pressing need for methods that can improve the image quality of neonatal images without increasing radiation exposure.

Several preprocessing techniques have been developed to enhance image quality, and clarity-based approaches have been widely explored. However, studies by Mei et al. ^3^ and Kats et al. ^4^ have shown that clarity-based methods can lead to a loss of detail or introduce artifacts, which can hinder accurate diagnosis. As a result, contrast-based techniques such as Histogram Equalization (HE) and Adaptive Histogram Equalization (AHE) have been proposed. Yet, these methods still have limitations in accurately enhancing lesion images. CLAHE (Contrast Limited Adaptive Histogram Equalization) has shown superior performance in adjusting contrast using clip-based contrast values. However, a major drawback of CLAHE is that it requires manual brute-force tuning of its hyperparameters, which is inefficient and has been highlighted as a limitation in previous studies. Although methods such as the entropy-based approach by B.S. Min et al. and LB-CLAHE by Gabriel Fillipe Centini Campos et al. have been proposed, they also face challenges in being universally applicable to all images.

**Figure 1:**
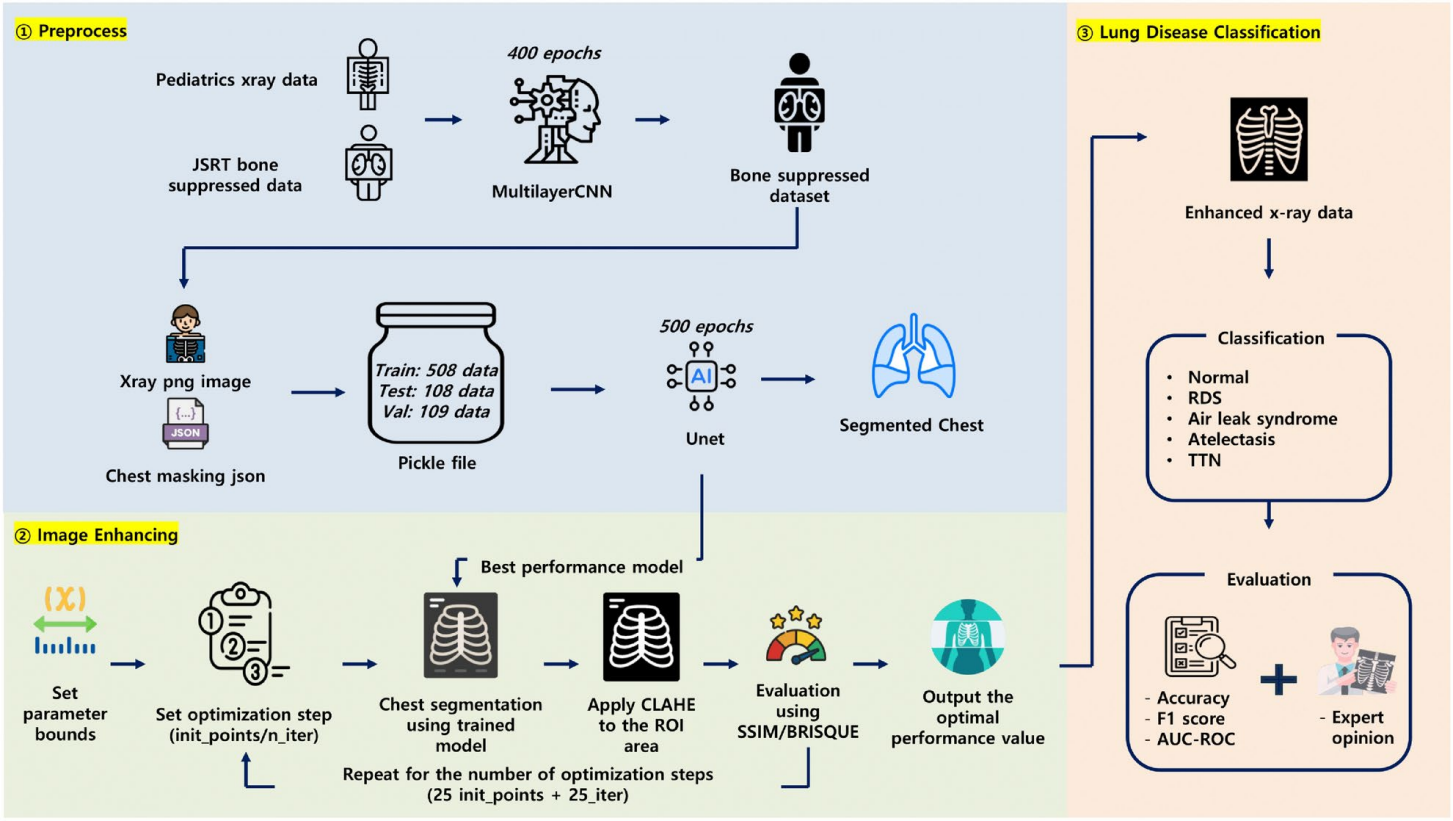
A comprehensive workflow for neonatal lung disease classification using BO-CLAHE-enhanced X-ray images. The process includes preprocessing with bone suppression and chest segmentation, image enhancement using Bayesian optimization, and final classification into five disease categories (Normal, RDS, Air Leak Syndrome, Atelectasis, and TTN) with evaluation based on accuracy, F1 score, AUC-ROC, and expert opinion.

To address these issues, this study presents the first proposal of a deep learning classification enhancement technique for lesion classification in preterm and high-risk neonates using BO-CLAHE. Prior to applying BO-CLAHE, bone suppression is used to emphasize lesions, and chest segmentation is applied to focus on the regions of interest. In the image enhancement stage, Bayesian optimization is utilized to automatically select the optimal hyperparameters for CLAHE, aiming to improve the image quality of preterm X-ray images (Fig. 1).

The evaluation results showed that images enhanced with BO-CLAHE significantly outperformed the original, unenhanced images in lesion classification. Particularly, there was a marked improvement in diagnosing Transient Tachypnea of the Newborn (TTN), a condition often confused with Respiratory Distress Syndrome (RDS) ^5^. From a pediatric perspective, clinicians reported that the enhanced images allowed for better visualization of anatomical structures and pathological features, enabling more precise assessments compared to traditional diagnostic evaluations. Furthermore, integrating deep learning models trained on these optimized images could accelerate the diagnostic process, potentially reducing the time needed for evaluation and interpretation. This time-saving aspect is crucial in treatment settings where timely intervention can significantly impact patient outcomes. This study represents a major advancement in the field, marking the first application of BO-CLAHE in neonatal lesion classification.

The contributions of this study can be summarized as follows:

- Reduced radiation exposure without compromising diagnostic quality.
- Automated hyperparameter tuning for CLAHE using Bayesian optimization.
- Proposal of an image enhancement method for AI-based lesion classification.
- Enhanced diagnostic accuracy.
- Timely and accurate pediatric diagnostic interventions.

## Results

### Overall performance evaluation

A key objective of this study is to facilitate rapid and accurate diagnosis of neonatal lung diseases. To achieve this, we conducted classification evaluations using four different models (GoogleNet, DenseNet161, ResNet18, Inception v3) on both original and BO-CLAHE enhanced images. As presented in Table 1, the BO-CLAHE enhanced images consistently demonstrated higher accuracy and F1 scores compared to the original images across most models. Specifically, the GoogleNet model showed a notable improvement in accuracy by 0.07 points. Particularly, the diagnosis of Transient Tachypnea of the Newborn (TTN) showed significant improvement.

**Table 1:** Performance comparison of classification models on enhanced and original data sets for 5-class classification.

Data	Evaluation metric	GoogleNet	DenseNet161	ResNet18	Inception V3
Enhanced	Accuracy	0.85	0.86	0.84	0.82
	F1 score	0.85	0.86	0.84	0.82
Original	Accuracy	0.78	0.85	0.82	0.83
	F1 score	0.78	0.85	0.82	0.83

As shown in Table 2, the evaluation was performed using multiple metrics, including accuracy, F1 score, precision, recall, and AUC, to provide a comprehensive assessment of the models’ performance. To ensure rigorous benchmarking, we compared BO-CLAHE-enhanced images with other preprocessing techniques such as BPDFHE and phycv, as well as with Real-ESRGAN, which employs a deep learning-based model for super-resolution enhancement. Among all methods, BO-CLAHE demonstrated superior performance across multiple evaluation metrics and classification models. Notably, the classification accuracy and F1 score for Transient Tachypnea of the Newborn (TTN)-a disease that is challenging to differentiate due to its visual similarities to Respiratory Distress Syndrome (RDS)-were significantly improved using BO-CLAHE-enhanced images. For instance, using the GoogleNet model, BO-CLAHE achieved an accuracy of 0.9 and an F1 score of 0.94 for TTN classification, outperforming BPDFHE (accuracy 0.8, F1 score 0.88), phycv (accuracy 0.7, F1 score 0.823), and Real-ESRGAN (accuracy 0.6, F1 score 0.8). This robustness was evident across other disease classes as well, with diseases having distinct visual features, such as Air Leak Syndrome and Atelectasis, being effectively identified with high accuracy and F1 scores across all models. Moreover, for visually complex and overlapping cases like TTN, BO-CLAHE consistently outperformed other methods, including Real-ESRGAN, which is designed for super-resolution rather than contrast enhancement. BO-CLAHE’s ability to improve diagnostic accuracy for TTN is particularly noteworthy, addressing a key challenge in distinguishing TTN from RDS and other visually similar conditions. This capability is critical in clinical applications where diagnostic precision directly impacts treatment outcomes. Overall, these findings underscore the effectiveness of BO-CLAHE as a preprocessing method that enhances image quality and significantly improves diagnostic performance across various disease categories and classification models.

**Table 2:** Performance comparison across models and preprocessing methods.

	Class	GoogleNet		DenseNet161		ResNet18		Inception_v3	
		Acc.	F1	Acc.	F1	Acc.	F1	Acc.	F1
Origin	Accuracy	0.81	–	0.85	–	0.82	-	0.83	-
	F1 score	-	0.81	-	0.85	-	0.82	-	0.83
	Air leak syndrome	0.71	0.81	0.8	0.89	0.75	0.86	0.8	0.89
	Atelectasis	0.75	0.853	0.8	0.89	0.75	0.86	0.9	0.95
	Normal	1.0	1.0	1.0	1.0	0.95	0.97	0.9	0.95
	RDS	0.95	0.97	1.0	1.0	1.0	1.0	0.95	0.97
	TTN	0.69	0.82	0.65	0.79	0.65	0.79	0.6	0.75
BPDFHE	Accuracy	0.82	-	0.78	-	0.8	-	0.82	–
	F1 score	-	0.81	-	0.779	-	0.799	-	0.814
	Air leak syndrome	0.7	0.92	0.75	0.857	0.8	0.888	0.85	0.85
	Atelectasis	0.8	0.88	0.8	0.888	0.85	0.918	0.725	0.75
	Normal	0.85	0.91	0.75	0.857	0.85	0.918	0.9	0.9
	RDS	0.95	0.97	0.95	0.974	0.9	0.947	1.0	1.0
	TTN	0.8	0.88	0.65	0.787	0.6	0.749	0.6	0.6
phycv	Accuracy	0.83	–	0.81	-	0.78	-	**0.83**	–
	F1 score	–	0.82	–	0.81	–	0.776	-	**0.828**
	Air leak syndrome	0.75	0.85	0.75	0.857	0.8	0.888	0.85	0.918
	Atelectasis	0.85	0.91	0.75	0.857	0.65	0.787	0.75	0.857
	Normal	0.95	0.97	0.8	0.888	0.8	0.888	0.8	0.888
	RDS	0.9	0.95	0.95	0.974	1.0	1.0	1.0	1.0
	TTN	0.7	0.82	0.8	0.888	0.65	0.787	0.75	0.857
Real-ESRGAN	Accuracy	0.825	-	0.809	-	0.809	-	0.79	-
	F1 score	-	0.827	-	0.811	-	0.812	–	0.796
	Air leak syndrome	0.785	0.898	0.785	0.88	0.714	0.833	0.785	0.88
	Atelectasis	0.8333	0.909	0.833	0.909	0.8333	0.909	0.75	0.857
	Normal	0.9	0.947	0.7	0.823	0.7	0.823	0.9	0.947
	RDS	0.9333	0.965	0.933	0.965	1.0	1.0	0.86	0.928
	TTN	0.6	0.8	**0.75**	**0.857**	0.75	0.857	0.6	0.8
BO-CLAHE	Accuracy	**0.86**	-	**0.86**	–	**0.84**	-	0.82	-
	F1 score	–	**0.86**	–	**0.86**	-	**0.84**	-	0.82
	Air leak syndrome	0.8	0.88	0.8	0.89	0.75	0.85	0.8	0.89
	Atelectasis	0.85	0.85	0.9	0.95	0.85	0.91	0.8	0.89
	Normal	0.8	0.89	0.9	0.95	0.85	0.91	0.7	0.82
	RDS	0.95	0.95	1.0	1.0	0.95	0.97	0.95	0.87
	TTN	**0.9**	**0.94**	0.7	0.82	**0.8**	**0.89**	**0.85**	**0.92**

### Statistical evaluation

**Figure 2:**
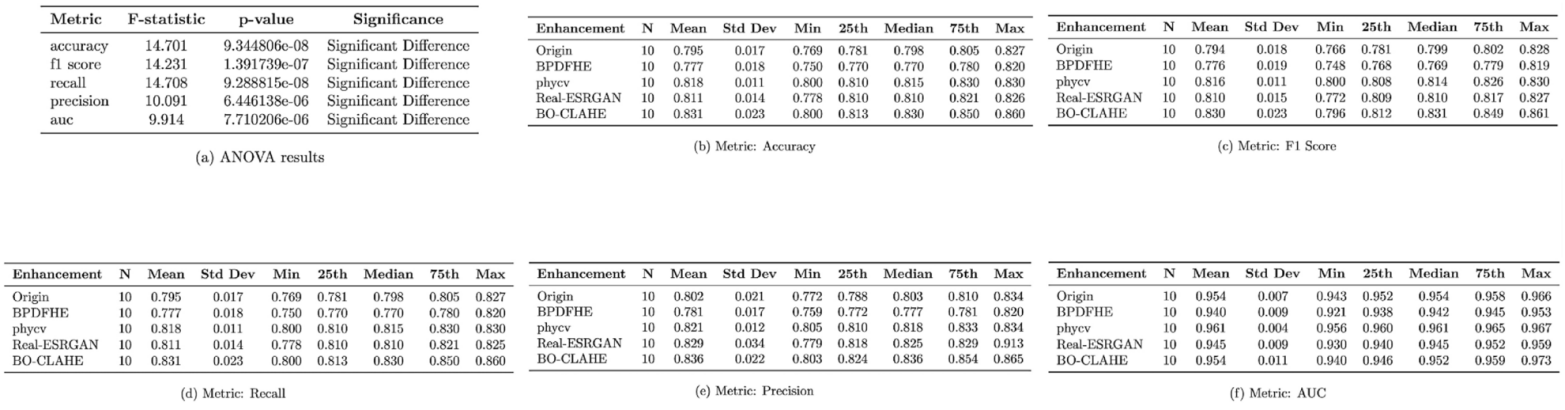
Statistical evaluation of accuracy, F1 score, recall, precision, and AUC for various enhancement methods. Table (**a**) summarizes ANOVA results, while Tables (**b–f**) provide descriptive statistics, including mean, standard deviation, and quartiles for each metric.

The statistical evaluation of accuracy, F1 score, recall, precision, and AUC based on the ANOVA results and descriptive statistics provided in Fig. 2 is detailed below.

#### Accuracy

The ANOVA results in Table (a) confirm a significant difference in accuracy among the methods ($F\text {-statistic} = 14.701$, $p\text {-value} = 9.3448 \times 10^{-8}$). According to Table (b), BO-CLAHE achieves the highest mean accuracy (0.831) with the smallest standard deviation (0.023), highlighting its stable and superior performance. In contrast, BPDFHE shows the lowest mean accuracy (0.777), while phycv (0.818) and Real-ESRGAN (0.811) demonstrate improvements over the original data (0.795).

#### F1 score

The ANOVA results in Table (a) show significant differences in F1 scores ($F\text {-statistic} = 14.231$, $p\text {-value} = 1.3917 \times 10^{-7}$). As shown in Table (c), BO-CLAHE achieves the highest mean F1 score (0.830) with a low standard deviation (0.023). Phycv (0.816) and Real-ESRGAN (0.810) improve upon the original data, but BPDFHE remains the lowest-performing method with a mean F1 score of 0.776.

#### Recall

Table (a) confirms statistically significant differences in recall across methods ($F\text {-statistic} = 14.708$, $p\text {-value} = 9.2888 \times 10^{-8}$). BO-CLAHE leads with the highest mean recall (0.831) and a standard deviation of 0.023, as shown in Table (d). Phycv (0.818) and Real-ESRGAN (0.811) show improvements over the original data (0.795), whereas BPDFHE achieves the lowest recall (0.777).

#### Precision

As indicated by Table (a), precision also shows significant differences ($F\text {-statistic} = 10.091$, $p\text {-value} = 6.4461 \times 10^{-6}$). Table (e) highlights BO-CLAHE as the best-performing method, with the highest mean precision (0.836) and the smallest standard deviation (0.022). Phycv (0.821) and Real-ESRGAN (0.829) outperform the original data (0.802) but exhibit higher variability. BPDFHE lags behind with the lowest precision (0.802).

#### AUC

Finally, Table (a) confirms significant differences in AUC among methods ($F\text {-statistic} = 9.914$, $p\text {-value} = 7.7102 \times 10^{-6}$). Table (f) shows that BO-CLAHE achieves the highest mean AUC (0.954) and a low standard deviation (0.011). While phycv (0.961) and Real-ESRGAN (0.945) improve upon the original data, BPDFHE records the lowest AUC (0.940).

### Visual analysis and clinical implications

**Figure 3:**
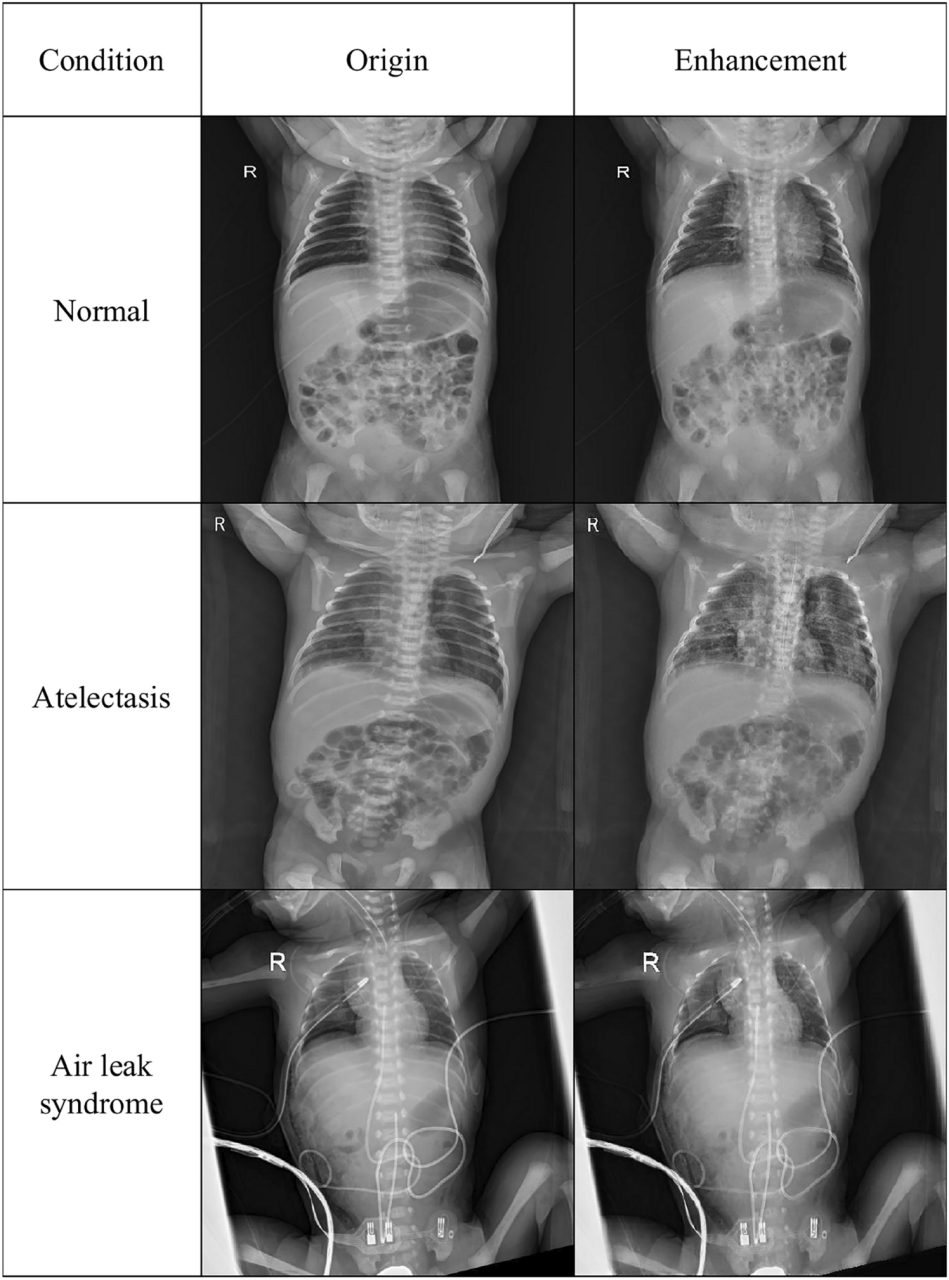
Comparison of X-ray images for different conditions with and without enhancement.

Beyond the numerical results, visual analysis further underscores the benefits of using BO-CLAHE enhanced images Fig. 3. A comparison of original and enhanced images reveals striking differences, especially in the chest area. The enhanced images provide much clearer and more distinct views of the organs and lesions in the chest, substantially contributing to diagnostic accuracy. This visual enhancement plays a vital role in medical imaging by helping healthcare professionals identify critical structures and lesions more accurately and efficiently, thus enabling faster and more informed decisions. The potential of image enhancement technology, like BO-CLAHE, to improve diagnostic precision is profound and highly promising.

Expert opinions from the neonatal care field further emphasize the importance of high-quality medical imaging for accurate disease diagnosis and assessment, especially in premature and high-risk neonates with respiratory issues. While X-ray imaging is widely used in these cases, its diagnostic value is heavily influenced by the clarity and quality of the images. Advanced image preprocessing techniques, such as noise reduction, contrast enhancement, and artifact removal, made possible by BO-CLAHE, allow clinicians to obtain optimized X-ray data. This leads to more accurate and informed evaluations, highlighting the critical role of enhanced imaging in neonatal care.

**Figure 4:**
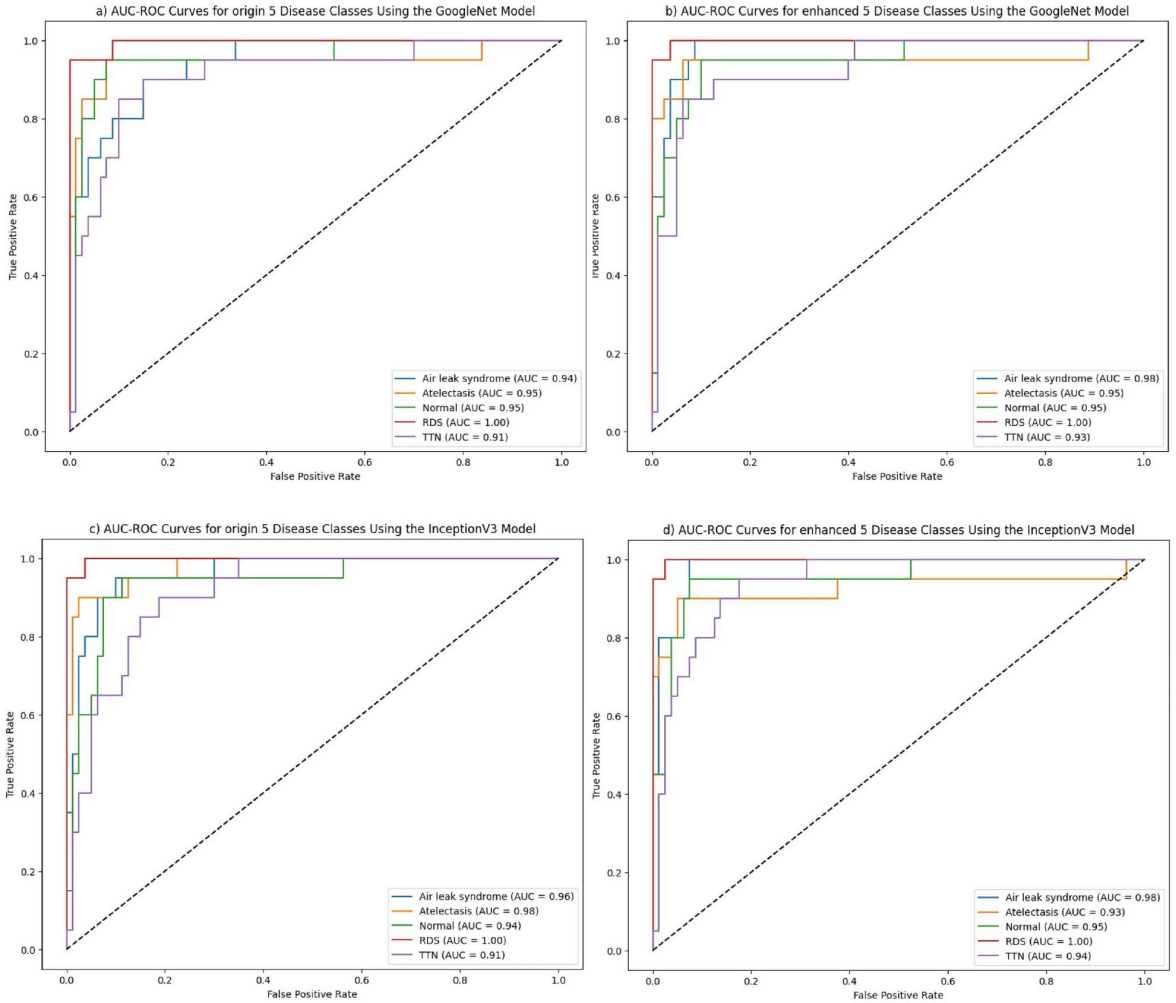
AUC-ROC curves for five disease classes using GoogleNet and InceptionV3 models on original and enhanced images.

The significance of this study is further illustrated in the AUC-ROC graphs Fig. 4. In the GoogleNet model results shown in sections a and b, a general increase in AUC values is observed, with a particularly notable improvement in the AUC value for TTN. Similarly, the results of the InceptionV3 model in sections c and d demonstrate an overall rise in AUC values for all classes, with a marked enhancement for TTN, showcasing the impact of BO-CLAHE enhanced images. These findings clearly demonstrate that the use of enhanced images significantly improves diagnostic accuracy across multiple models.

### Preprocessing evaluation

**Table 3:** Performance comparison of different data preprocessing methods.

Data	Metric	Air leak syndrome	Atelectasis	Normal	RDS	TTN
Origin	Accuracy	0.6	0.85	0.8	0.95	0.7
	F1 score	0.75	0.92	0.89	0.97	0.82
Only boneless (without CLAHE)	Accuracy	0.7	0.8	0.85	0.95	0.72
	F1 score	0.82	0.89	0.91	0.97	0.84
Only CLAHE (without mask)	Accuracy	0.78	0.8	0.75	1.0	0.6
	F1 score	0.87	0.88	0.85	1.0	0.75
BO-CLAHE	Accuracy	0.9	0.85	0.8	0.95	0.75
	F1 score	0.95	0.92	0.89	0.97	0.86

Bone suppression and chest segmentation are critical preprocessing steps that significantly enhance the detection and classification of lesions by refining the quality and focus of the input data. As summarized in Table 3, the inclusion of bone suppression, which removes high-density structures like ribs that obscure soft tissue details, improves performance metrics. For instance, applying only bone suppression increases the accuracy and F1 score for Air Leak Syndrome from 0.6 and 0.75 to 0.7 and 0.82, respectively. Similarly, chest segmentation isolates diagnostically relevant regions, ensuring that enhancement techniques focus on areas of clinical importance while reducing the influence of irrelevant features. Without segmentation masks, the performance gains from CLAHE alone are limited, as seen in TTN with an accuracy of 0.75 and an F1 score of 0.75.

The full BO-CLAHE pipeline, as presented in Table 3, which integrates bone suppression, chest segmentation, and CLAHE optimized with Bayesian methods, achieves superior results across all disease categories. Notably, TTN classification improves substantially, with accuracy reaching 0.85 and the F1 score increasing to 0.92. These findings highlight the indispensable role of bone suppression and chest segmentation in preprocessing, enabling better enhancement of lesion-specific features and maximizing diagnostic performance.

#### Bone suppression

In the diagnosis of lung diseases, the primary focus is on the lungs and surrounding organs, while the spine and ribs play a less critical role. However, for the accurate diagnosis of various conditions, the spine and ribs can serve as useful reference points. Despite this, removing the bones can significantly enhance diagnostic efficiency by emphasizing lung-related structures, leading to more precise classification and better detection of lesions. By suppressing the bones in X-ray images, we can achieve a clearer view of the lungs and associated organs, which is essential for improving diagnostic outcomes in lung diseases.

**Figure 5:**
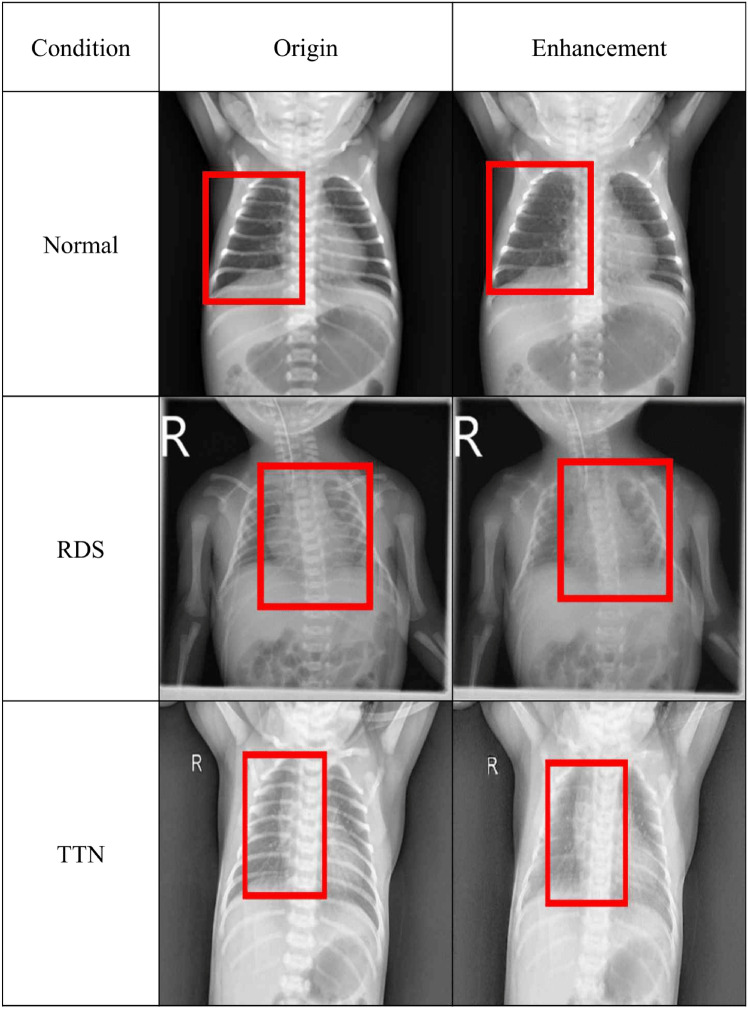
Comparison of original and enhanced X-ray images after bone removal for different conditions.

For training the bone suppression process on X-ray images, bone-free images of neonatal patients are ideal. However, due to challenges such as limiting radiation exposure, it is more practical to use bone suppression images from the JSRT dataset, which contains adult images. The results of this process are shown in Fig. 5. A comparison between the original and bone-suppressed images reveals that the spine and ribs surrounding the heart and lungs are substantially lightened or removed. Importantly, the anatomical structures of the organs behind the bones are well-preserved, allowing for easier identification of lesions and improved diagnostic clarity.

#### Chest segmentation

**Figure 6:**
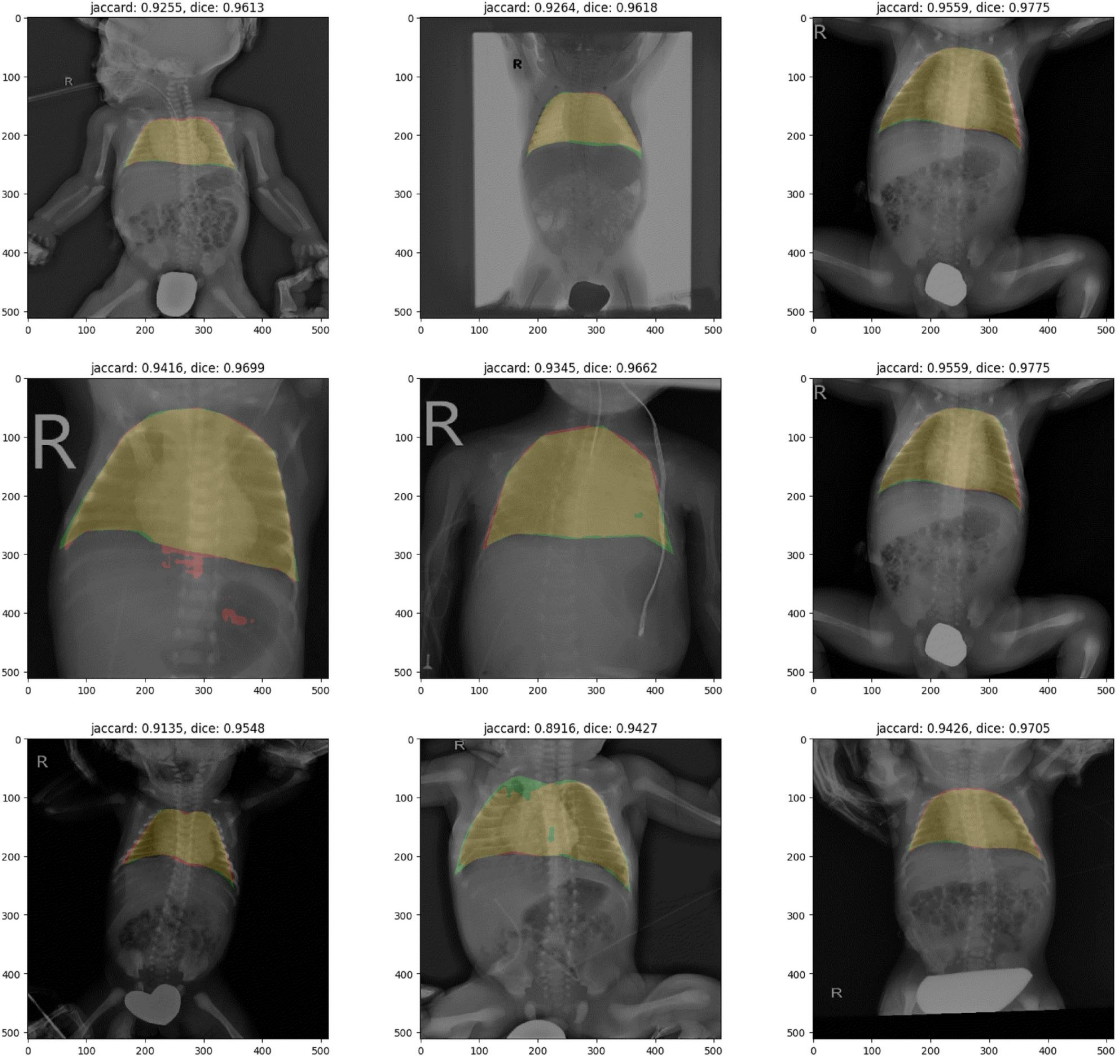
Chest segmentation result.

This study focuses on neonatal lung-related diseases. When applying CLAHE to the entire image, information from non-chest areas may be included, potentially affecting the results. To mitigate this, an experiment was designed to segment only the chest area before applying CLAHE. This approach yielded an average test loss of 0.031, an average Jaccard index of 0.88, and an average Dice coefficient of 0.93. As shown in Fig. 6, the red area represents the predicted region, the green area represents the ground truth, and the yellow area indicates the intersection of the two. The predicted and ground truth areas align closely, resulting in a predominantly yellow output. When experimenting with new image data for each disease using the trained model, it was confirmed that segmentation yielded highly accurate results.

## Discussion

In neonatal radiology, particularly for premature and high-risk neonates, accurate diagnosis and effective treatment of respiratory diseases are critical. X-ray imaging serves as a fundamental diagnostic tool in this field, where the quality and clarity of images are essential for diagnosing conditions like Transient Tachypnea of the Newborn (TTN) and Respiratory Distress Syndrome (RDS), which often present with similar symptoms.

Our research demonstrates significant advancements by implementing BO-CLAHE, an advanced image preprocessing technique, in conjunction with deep learning models. These methods have proven to enhance diagnostic accuracy significantly. Neonatal radiologists participating in our study noted that BO-CLAHE-enabled image enhancement provides superior visualization of subtle anatomical features, which is crucial for distinguishing between conditions such as TTN and RDS.

The use of deep learning models trained on BO-CLAHE enhanced images marks a breakthrough approach. By capturing the nuanced differences in preprocessed images, these models improve the ability to differentiate between conditions that require distinct treatment protocols. Misdiagnosis in these cases can lead to inappropriate treatment, which could result in severe or life-threatening complications in neonates.

Furthermore, this study developed optimized preprocessing algorithms and deep learning frameworks specifically designed for neonatal X-ray imaging. These innovations contribute significantly to more reliable and faster assessments of respiratory diseases in neonates, which is essential in critical care environments where quick decision-making is crucial for patient outcomes.

On the technical front, our exploration of bone suppression techniques involved both original and bone-suppressed images, which are necessary for analyzing the removal of bones in X-ray images. Adjusting radiation doses to obtain bone-free X-ray images can be risky for neonates due to their higher sensitivity to radiation. Therefore, we utilized adult JSRT data to mitigate this risk. The potential future development of bone suppression technology that does not rely on ground truth data could greatly enhance medical imaging.

The application of BO-CLAHE and other advanced techniques not only improves diagnostic precision but also reduces the time required for accurate interpretation-an advantage of paramount importance in critical care settings, where rapid decision-making can have a dramatic impact on clinical outcomes.

## Methods

### Research design

This study analyzed data collected by universities and hospitals, including Korea University, using ’Pediatric Abdominal X-ray Image Data’. The study focused on X-ray images taken for medical purposes, particularly those of neonatal patients weighing less than 10 kg or more than 30 kg. Data collection was conducted in cooperation with each institution, and only data suitable for the objectives and needs of the research were selected for use.

#### Sample size and sampling technique

In this study, a total of 17,434 cases were used for analysis, with approximately 6,133 cases belonging to the normal group and 11,301 cases to the disease group. The disease group consisted of 2,352 cases of air leak syndrome, 1,674 cases of atelectasis, 3,001 cases of respiratory distress syndrome (RDS), and 4,274 cases of transient tachypnea of the newborn (TTN). The dataset used for analysis was selected from an initial pool of approximately 67,000 images, from which the top 17,434 high-quality images, free from unnecessary elements, were chosen. The selected images were devoid of obstructions such as scissors or the hands of parents or guardians, and had minimal unnecessary margins and minimal blurring. Each institution established data selection criteria based on these characteristics, contributing to the overall accuracy and precision of the study.

#### Data construction and utilization

The collected dataset consisted of image files in .png format, json files, and dicom files; however, this study utilized only the image files and json annotations. The json annotations included a variety of information such as the openness of polygons, area, and object coordinates, with particular emphasis on the segmentation of the lung area based on labeling type, category, and object coordinate information. This approach markedly enhanced the precision of the research and maximized the utilization of the data.

#### Data collection and IRB approval

Portable supine chest X-ray images for 6 classes of healthy normal lung, respiratory distress syndrome (RDS), transient tachypnea of the newborn (TTN), air leak syndrome (ALS), atelectasis, and bronchopulmonary dysplasia (BPD) were retrospectively collected from the NICUs of 10 university hospitals in Republic of Korea from January 2020 to December 31, 2022. This registry was approved by the institutional review board (IRB) at each participating hospital. The IRB granted a waiver of informed consent due to the study’s retrospective design, the absence of personal information, and its meticulous execution under the guidance of the institute’s ethics committee. The research was conducted in accordance with IRB protocol (IRB-2022AS0056).

### The specifications of the hardware and software used in the research

This study utilized a specific set of hardware and software components to support the research activities. The operating system implemented was Linux version 5.15.0-102-generic. The server’s processing capabilities were provided by 36 CPU cores. Graphical computations were managed using two NVIDIA GeForce RTX 4090 GPUs. The development environment comprised CUDA version 12.0, Python version 3.11.0, PyTorch version 2.2.1, and NumPy version 1.26.3, forming a comprehensive computational framework essential for the research.

### BO-CLAHE

The BO-CLAHE algorithm is an advanced image enhancement approach designed to optimize neonatal chest X-ray images for accurate diagnosis. It combines bone suppression, chest segmentation, and Contrast Limited Adaptive Histogram Equalization (CLAHE) with Bayesian optimization. The process starts by suppressing bone structures using a convolutional neural network trained on JSRT bone-suppressed datasets to highlight soft tissues. Subsequently, a U-Net model is employed to segment chest areas, focusing on regions critical for diagnosis. The core of the method involves fine-tuning CLAHE’s hyperparameters-clipLimit and tileGridSize-using Bayesian optimization within a predefined parameter space. By iteratively evaluating the Structural Similarity Index (SSIM), the algorithm ensures precise image enhancement while preserving diagnostically significant details. These enhanced images demonstrate substantial improvements in lesion visibility, providing a reliable foundation for downstream applications, including automated lesion classification and disease diagnosis.

**Algorithm 1:**
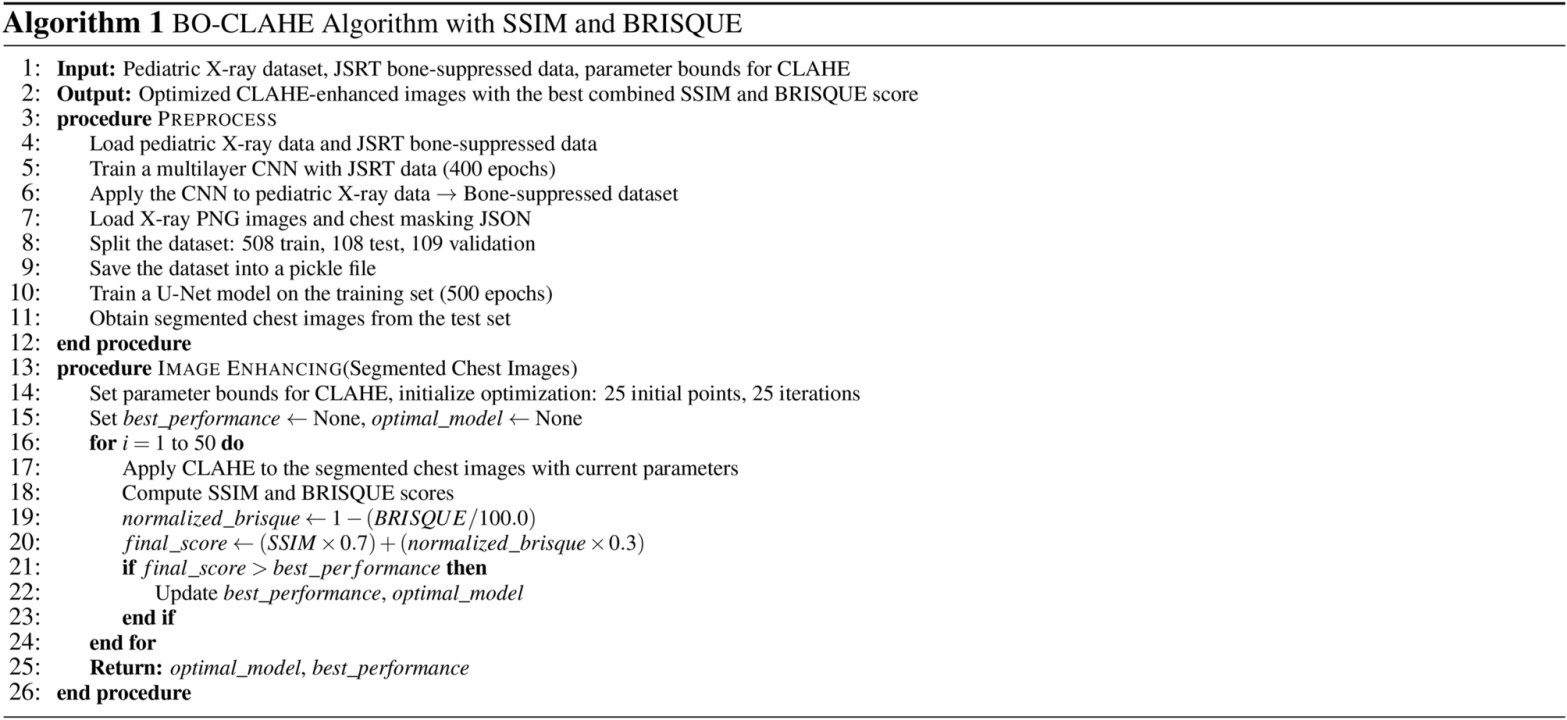
BO-CLAHE algorithm with SSIM and BRISQUE

### Bone suppression

Bone suppression technology is crucial in chest X-ray imaging for reducing obstructions caused by bone structures like ribs and vertebrae, thereby enhancing the visibility of soft tissues and early signs of lung lesions. Previous research, such as the work by P. Shah ^6^, has shown that removing bone structures can improve diagnostic performance for lung cancer. Similarly, Gusarev et al. developed a multi-layer convolutional neural network (CNN)-based bone suppression model ^7^, which has been instrumental in improving the accuracy of diagnosing tuberculosis, pneumonia, and lung cancer.

In this study, we advanced these models by applying an enhanced deep learning-based bone suppression technique to minimize the impact of bones in chest X-ray images and better reveal lung tissue details. The GusarevModel.MultilayerCNN model^7^, implemented in PyTorch and trained on the JSRT dataset, was used to generate bone-suppressed images. Custom transformations and a DataLoader were employed to manage the image processing. After resizing and converting each image to a tensor, the model produced bone-suppressed outputs, which were resized to the original dimensions and saved.

This process effectively suppresses bones in chest X-ray images, contributing to improved diagnostic accuracy for lung diseases.

### CLAHE

CLAHE (Contrast Limited Adaptive Histogram Equalization) is an image processing technique that uses histograms to adjust contrast in images. The traditional method of Histogram Equalization (HE) adjusts contrast using the histogram of the entire image, but it can amplify noise in specific areas. To address this issue, Adaptive Histogram Equalization (AHE) was introduced, which divides the image into small rectangular tiles and applies histogram equalization to each tile. However, AHE can also amplify noise in areas with concentrated pixel intensity. Developed to solve these issues, CLAHE follows the principle of AHE but introduces a technique to limit the maximum height of histograms, reducing noise amplification and enhancing overall image quality.

Among various methods to improve the quality of X-ray images, CLAHE is actively used. However, a clear method for selecting its hyperparameters, such as clipLimit and tileGridSize, has not yet been established. B. S. Min and colleagues ^8^ proposed a method to automatically determine hyperparameters based on entropy, identifying CL and NT values by locating the point of maximum curvature on the entropy curve. Additionally, Gabriel Fillipe Centini Campos and others ^9^ introduced a learning-based hyperparameter selection method called LB-CLAHE, which utilizes an ensemble model derived from RF and XGBoost algorithms. This study showed excellent performance, but it pointed out that some adjustments are needed to successfully apply it to specific computer vision applications.

In this study, the parameter ranges for clipLimit and tileGridSize were selected based on values commonly used in previous research on traditional CLAHE and LB-CLAHE methods. Specifically, the range for clipLimit was set to (1.0, 255.0), and for tileGridSize to (8.0, 32.0). These ranges were chosen to encompass typical values observed in similar applications, ensuring robust optimization while avoiding over-enhancement or under-enhancement. Given the complexity of manually tuning these parameters, Bayesian Optimization was employed to efficiently explore and identify the optimal values. SSIM (Structural Similarity Index) was used as the evaluation metric during optimization to ensure that the enhanced images closely resembled the original images in terms of structural details and contrast. Additionally, as highlighted in the paper Perceptual Video Quality Assessment: A Survey ^10^, the limitations of relying solely on SSIM were addressed by integrating BRISQUE (Blind/Referenceless Image Spatial Quality Evaluator), a no-reference image quality assessment metric. This dual-metric approach ensures a more comprehensive evaluation of both structural fidelity and perceptual quality. The optimized parameters were validated by comparing the classification performance of images processed with these parameters across multiple deep learning models.

### Chest segmentation

For chest segmentation, a total of 723 images were collected and segmented using masking techniques ^11^. The dataset was split into training, validation, and test sets in a 70:15:15 ratio. Each image was resized to 512x512 pixels for standardization as input to the model. The U-Net architecture, widely used in the medical imaging field, was employed for this task, and the model was implemented using the PyTorch framework.The training data consisted of chest X-ray images and their corresponding mask images. The images were converted into tensors and resized, and a DataLoader was used to efficiently handle batches of data during training. The Adam optimizer was used with a learning rate of 0.0003. The model was trained over 500 epochs, with performance metrics such as loss, Jaccard index, and Dice coefficient used to monitor the training process.During the validation phase, the model was evaluated on the validation dataset at each epoch. The Jaccard index and Dice coefficient were calculated to measure the similarity between the predicted segmentation and the ground truth. The model with the lowest validation loss was saved. Upon completion of training, the model achieved an average test loss of 0.0308, a Jaccard index of 0.8827, and a Dice coefficient of 0.9306 on the test dataset. As shown in Fig. 6, the results were visualized by overlaying the predicted segmentation on the original images, allowing for a visual comparison between the ground truth and the model’s predictions.

### Bayesian optimization

Bayesian Optimization was developed to address the limitations of traditional hyperparameter optimization methods like grid search and random search. Grid search exhaustively explores all hyperparameter combinations, which is computationally expensive in high-dimensional spaces ^12^. Random search improves efficiency by sampling combinations randomly but may miss optimal solutions due to its lack of systematic exploration ^13^.To overcome these issues, Bayesian Optimization uses a surrogate model to estimate objective function values and an acquisition function to guide exploration. This method efficiently targets areas with high uncertainty or potential, offering less bias than random or grid search and performing well even under fairness constraints or robust extensions ^14^.

To ensure systematic optimization, the hyperparameter ranges and settings used in this study for Bayesian Optimization are summarized in Table 4. These configurations were designed to balance exploration and exploitation effectively, enabling an efficient search for optimal CLAHE parameters.

**Table 4:** Hyperparameter ranges and Bayesian optimization settings.

Parameter	Value	Description
Search space	clipLimit (1.0, 255.0)	Range for the CLAHE clip limit parameter
	tileGridSize (8.0, 32.0)	Range for the tile grid size parameter for CLAHE
Initial sampling	init_points = 25	Number of random samples before the optimization starts
Optimization iterations	n_iter = 25	Number of iterations for Bayesian Optimization
Exploration vs exploitation	UCB constant kappa (2.576)	Upper confidence bound constant for exploration-exploitation trade-off
	EI constant xi (0.01)	Expected improvement constant for exploration sensitivity
Gaussian process settings	Noise level alpha ($1 \times 10^{-6}$)	Noise level to account for stochasticity in observations
	Restarts for optimizer (5)	Number of restarts to avoid local optima during kernel optimization
Target normalization	normalize_y = True	Whether to normalize target values to improve optimization performance

These configurations enabled a systematic and efficient exploration of the hyperparameter space. The search space for CLAHE parameters included clipLimit, controlling the contrast clipping threshold, and tileGridSize, determining the size of local regions for histogram equalization. The optimization process began with an initial random sampling phase (init_points = 25), which provided a robust starting dataset for the optimization. This was followed by n_iter = 25 iterations of Bayesian Optimization to iteratively refine the parameters. Trade-offs between exploration and exploitation were guided by the Upper Confidence Bound (UCB) constant kappa, set to 2.576, and the Expected Improvement (EI) constant xi, fixed at 0.01. These settings balanced the need for exploring uncertain areas and exploiting known promising regions. Gaussian Process optimization was stabilized using a noise level alpha of $1 \times 10^{-6}$ and five restarts for the optimizer to avoid convergence to local optima. Additionally, normalization of the target values (normalize_y = True) ensured improved convergence by standardizing the target value range.

In this study, Bayesian optimization was employed to efficiently optimize the hyperparameters of CLAHE (Contrast Limited Adaptive Histogram Equalization) with the goal of enhancing the quality of X-ray images. The key evaluation functions used were the Structural Similarity Index (SSIM) and Blind/Referenceless Image Spatial Quality Evaluator (BRISQUE). *SSIM* is a metric that evaluates the similarity between two images by assessing correlation loss, brightness distortion, and contrast distortion. A higher SSIM value, closer to 1, indicates greater similarity between the images. The SSIM is calculated as follows:

$$SSIM(x, y) = l(x, y)^\alpha \cdot c(x, y)^\beta \cdot s(x, y)^\gamma$$

*BRISQUE*, on the other hand, is a no-reference image quality metric that quantifies the perceptual quality of an image based on natural scene statistics. Unlike SSIM, which compares two images, BRISQUE assesses a single image to identify distortions. A lower BRISQUE score indicates better perceptual quality, with values closer to 0 representing images of higher quality. This approach ensured that the optimization process accounted for both structural similarity to the original image and standalone perceptual quality improvements. By combining SSIM and BRISQUE, the Bayesian optimization process effectively identified the optimal CLAHE parameters for enhancing the quality of X-ray images.

The optimization process focused on segmenting the lung region in neonatal chest X-ray images and subsequently applying CLAHE to improve the image quality. The procedure began by loading a pre-trained U-Net model, configuring the computational environment GPU, and processing the original grayscale images to identify the lung region. CLAHE was then applied to the segmented lung area, with the hyperparameters, such as clipLimit and tileGridSize, defined and applied. The quality of the processed images was evaluated by comparing them to the original images using the SSIM, BRISQUE metric. During the optimization process, the range of CLAHE hyperparameters was defined, and the evaluation function partially incorporated image paths. The goal was to maximize the SSIM, BRISQUE score, identifying the set of CLAHE parameters that best enhanced the similarity between the original and enhanced images. Ultimately, the optimized CLAHE parameters were applied to the original images, and the enhanced images were saved in a designated output directory. Bayesian optimization efficiently explored the hyperparameter space, iteratively adjusting and identifying the optimal parameters to maximize image quality.

**Figure 7:**
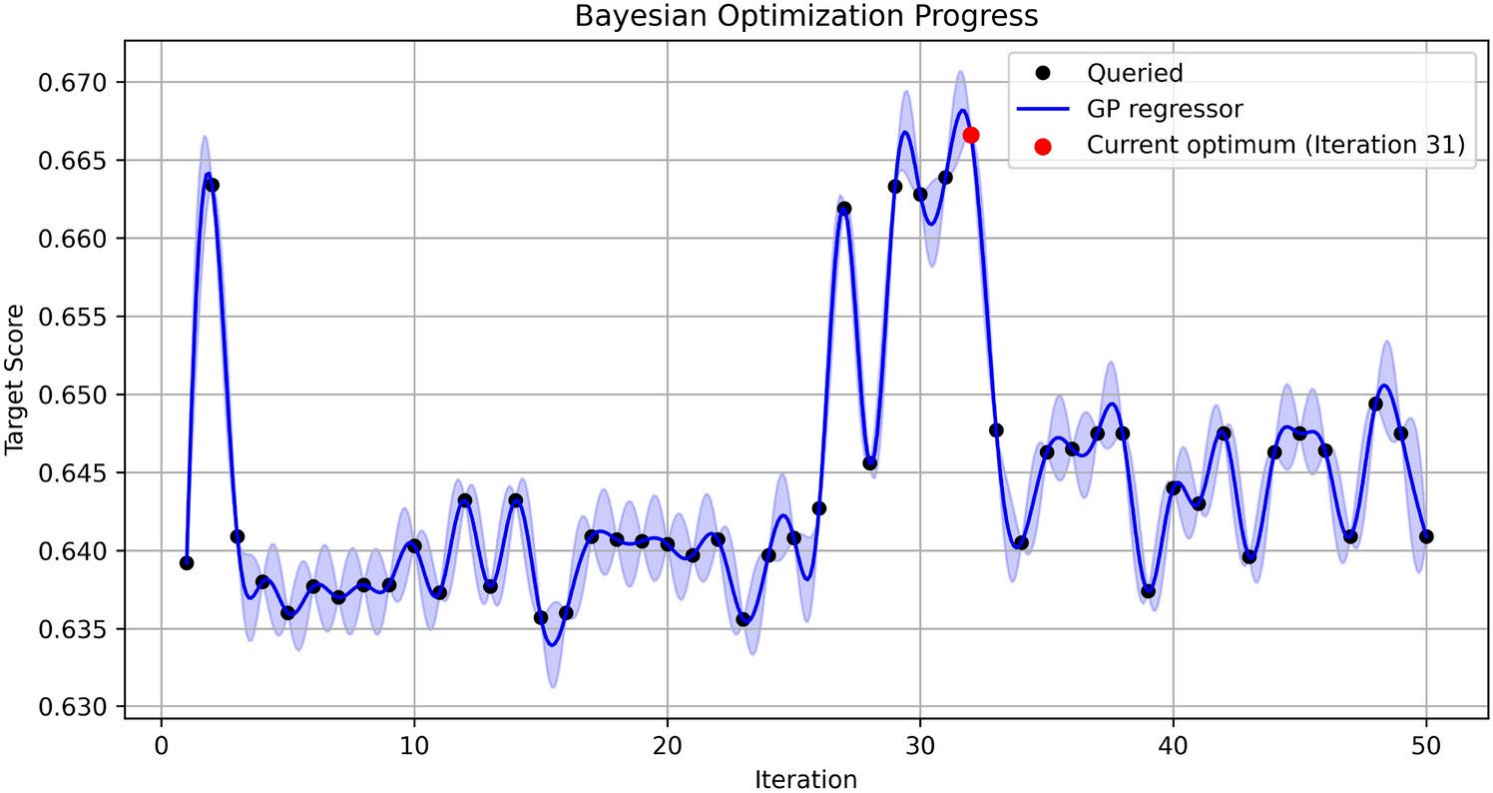
Bayesian optimization.

The graph in Fig. 7 visually illustrates the progress of Bayesian optimization. Each iteration represents the evaluation of different hyperparameter combinations for the CLAHE algorithm. The y-axis shows the target score for each iteration, while the x-axis indicates the number of iterations.

1. Black dots (queried points) Each black dot represents the result of evaluating a specific hyperparameter combination for CLAHE. These points reflect the SSIM, BRISQUE values obtained during each iteration, based on the hyperparameters selected by Bayesian optimization.
2. Blue line (GP regressor prediction) The blue line shows the prediction of the Gaussian Process (GP) regression model used in Bayesian optimization. This line represents the GP’s estimation of the relationship between the hyperparameters and the target score, and it is continuously updated throughout the optimization process.
3. Blue shaded area (95 confidence interval) The blue shaded area represents the uncertainty in the GP’s predictions, indicating a 95 confidence interval. Areas of higher uncertainty are shown with wider shading. Bayesian optimization takes this uncertainty into account when selecting the next region to explore.
4. Red cross (current best point) The red cross indicates the highest SSIM, BRISQUE score obtained so far, representing the best combination of hyperparameters identified during the optimization process

By utilizing Bayesian optimization, the hyperparameters of CLAHE were efficiently optimized, improving the quality of chest X-ray images of low birth weight neonatals. The surrogate model, based on a Gaussian Process, predicted the relationship between hyperparameters and the SSIM, BRISQUE score, while the acquisition function selected the next point to explore. This process enabled the evaluation of new hyperparameter combinations in each iteration, ultimately identifying the parameters that maximized the SSIM, BRISQUE score. Bayesian optimization demonstrated its efficiency by finding optimal parameters with fewer iterations, effectively enhancing image quality.

The BO-CLAHE algorithm integrates advanced preprocessing techniques and optimization methods to enhance neonatal chest X-ray images effectively. By incorporating bone suppression, chest segmentation, and Bayesian optimization, it achieves high-quality, diagnostically relevant image enhancement. This approach not only improves lesion visibility but also minimizes manual parameter tuning, making it an indispensable tool in neonatal radiology. The algorithm’s ability to optimize CLAHE parameters systematically ensures consistent diagnostic quality, facilitating more precise identification of conditions such as Transient Tachypnea of the Newborn (TTN). Moreover, the integration of enhanced imaging with deep learning models accelerates the diagnostic process, paving the way for timely interventions and improved patient outcomes in neonatal care.

## Conclusion

This study highlights the critical importance of advanced diagnostic techniques in the treatment of respiratory diseases in premature and high-risk neonates. By integrating sophisticated image preprocessing methods, such as BO-CLAHE, with deep learning models, we significantly enhanced the quality and diagnostic effectiveness of neonatal X-ray imaging. The application of BO-CLAHE and Bayesian optimization played a key role in improving the clarity of vital medical information within the images, leading to better diagnostic accuracy. In particular, the enhanced images showed remarkable efficacy in the classification of Transient Tachypnea of the Newborn (TTN).

Moreover, these technological advancements enable more accurate differentiation of visually similar diseases. The incorporation of deep learning models trained on these optimized images further accelerates the diagnostic process, shortening the time needed for evaluation and interpretation. Clinicians have observed that these improvements translate into faster and more precise diagnoses.

Looking forward, future research will expand beyond X-ray imaging to integrate clinical data for more comprehensive diagnostic analysis. Additionally, longitudinal studies utilizing AI-based diagnostic technologies, such as BO-CLAHE, are planned to further optimize diagnostic procedures and improve outcomes in neonatal care. This research sets the foundation for new standards in neonatal medicine, offering the potential to elevate the quality of care and establish benchmarks in neonatal healthcare.

## Figures and Tables

**Figure 8:**
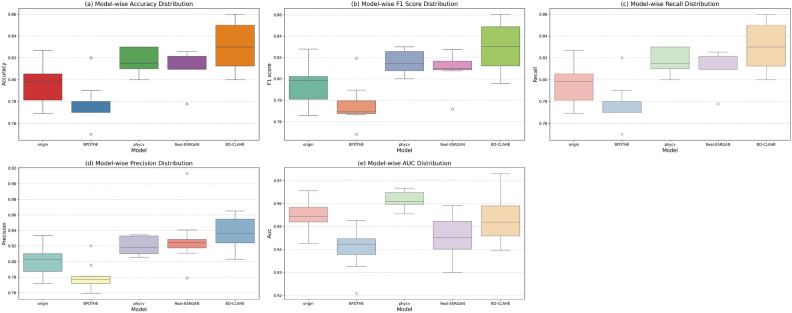
Boxplot distributions of model-wise performance metrics, including (**a**) accuracy, (**b**) F1 score, (**c**) recall, (**d**) precision, and (**e**) AUC. Each plot compares the performance of different enhancement methods: origin, BPDFHE, PhyCV, Real-ESRGAN, and BO-CLAHE.

## Data Availability

The data supporting the findings of this study are not publicly available due to privacy concerns and the protection of personal medical information. However, data may be available from the corresponding author upon reasonable request, subject to appropriate ethical and legal considerations

## Appendix

### ANOVA boxplot distributions of model-wise performance metrics

The boxplots in Fig. 8 provide a visual comparison of performance metrics (Accuracy, F1 Score, Recall, Precision, and AUC) across different enhancement methods. The following insights can be drawn from the visualizations.

#### Accuracy

The boxplot for accuracy highlights BO-CLAHE’s superior and consistent performance, with the highest median accuracy and the narrowest interquartile range. In contrast, BPDFHE shows the lowest accuracy with significant variability, while phycv and Real-ESRGAN perform better than the original data but remain below BO-CLAHE.

#### F1 score

The F1 score distribution illustrates BO-CLAHE’s ability to maintain a balanced performance between recall and precision, achieving the highest median F1 score. BPDFHE consistently underperforms, while phycv and Real-ESRGAN demonstrate moderate improvements over the original data.

#### Recall

BO-CLAHE achieves the highest recall across all models, emphasizing its capability to correctly identify true positives. BPDFHE shows the lowest recall with wide variability, reflecting inconsistent results.

#### Precision

The precision boxplot shows that BO-CLAHE minimizes false positives effectively, achieving the highest median precision. Phycv and Real-ESRGAN exhibit variability, indicating less consistent performance across models.

#### AUC

BO-CLAHE demonstrates the highest median AUC, reflecting its robustness in distinguishing between disease classes. The narrow interquartile range underscores its reliability, while BPDFHE shows the lowest AUC with significant variability.
